# Treatment of Pyoderma Gangrenosum With Mycophenolate and Hyperbaric Oxygen Therapy: A Case Report and Literature Review

**DOI:** 10.7759/cureus.38159

**Published:** 2023-04-26

**Authors:** Subo Dey, Nirali Sanghavi, Amy Wasserman, Kausik Kar

**Affiliations:** 1 Internal Medicine, Westchester Medical Center, Valhalla, USA; 2 Rheumatology, Westchester Medical Center, Valhalla, USA; 3 Internal Medicine, Wound and Hyperbaric Medicine, Westchester Medical Center, Valhalla, USA

**Keywords:** oral methotrexate, prednisone treatment, hyperbaric oxygen thetapy, mycophenolate mofetile, pyoderma gangenosum

## Abstract

Pyoderma gangrenosum is an uncommon inflammatory ulcerative skin disorder with an unclear etiology. In many cases, it is associated with several underlying systemic diseases, with inflammatory bowel disease being the most common one. Since it does not have any specific clinical or laboratory findings, it is a diagnosis of exclusion. A multidisciplinary approach is vital in treating pyoderma gangrenosum. Its recurrence remains common, and it also has an unpredictable prognosis. Here, we report a case report of pyoderma gangrenosum, which was successfully treated with mycophenolate and hyperbaric oxygen therapy.

## Introduction

Pyoderma gangrenosum (PG) is an uncommon inflammatory and ulcerative skin disorder induced by neutrophils and inflammatory cytokines with an unclear etiology [1]. PG was first described by Brocq in 1916 [2], but the term "pyoderma gangrenosum" (PG) was first coined by Brunsting et al. in 1930 [3]. Many systemic diseases, such as inflammatory bowel disease, rheumatologic conditions, and hematologic disorders (immunoglobulin A (IgA) monoclonal gammopathy, myelodysplasia), have been described as being associated with PG [4]. There is no gold standard for the treatment of PG. At first, PG was thought to be associated with infection; however, over the next 85 years, it was concluded that ulcers due to PG were primarily sterile with periodic secondary colonization [3]. Most commonly, PG develops on the lower extremities, often associated with minor trauma, but it can occur anywhere, including surgical sites [5].

## Case presentation

We present the case of a 56-year-old gentleman with a smoking history of 10 packs per year, an additional history of hypertension, and a biopsy-diagnosed pyoderma gangrenosum (PG). He presented to our hospital in May 2021 with the worsening of his chronic left lower extremity necrotic wound secondary to his PG. The wound appeared around April 2020 as a small scab in his left lower extremity, and it slowly developed into a necrotic wound. Subsequently, he had multiple necrotic areas around the tibial region along with severe pain. He denied systemic symptoms of fever or chills. Before the patient was diagnosed with PG, he was treated with multiple antibiotics along with topical steroids without any significant improvement. He was also given mupirocin and used topical hydrogen peroxide, along with 10 mg of oral prednisone, for a brief period. He later underwent a skin biopsy that was consistent with PG.

**Figure 1:**
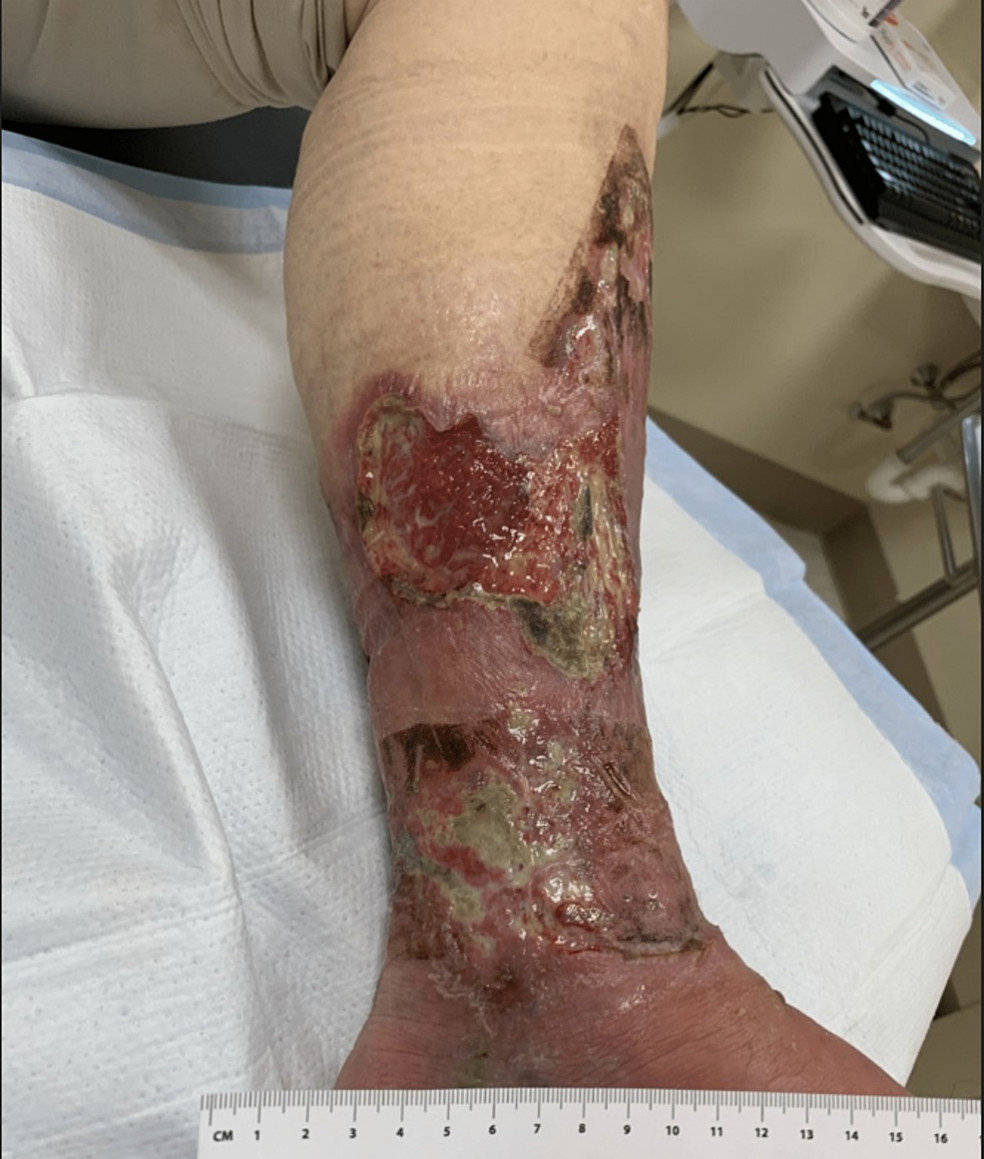
The wound during the patient's initial presentation to the wound clinic in July 2021

During his first hospitalization in May 2021, no surgical intervention was performed; he consistently followed up with outpatient wound care services. Subsequently, the patient started receiving hyperbaric oxygen treatment in August 2021 for his deteriorating wounds (at least four discrete wounds) in the left lower extremity, left medial leg (4.0 cm x 3.5 cm x 0.3 cm), left anterior medial leg (5.5 cm x 5.0cm x 0.3 cm), and left lateral leg. The wounds were all described as chronic, full-thickness vasculitis ulcers. The patient's initial presentation of the wound is depicted in Figure 1.

Initial blood work showed that the patient was negative for antinuclear antibody (ANA), anti-neutrophil cytoplasmic antibody (ANCA), hepatitis C antibody, hepatitis B serology, HIV antigen or antibody, and rheumatoid factor (RF). His urinalysis noted 1+ protein and no red blood cells. During his subsequent hospitalization in October 2021, the wound culture and blood culture did not yield any positive results. Hepatitis B and hepatitis C were also ruled out as potential etiologies of the wounds, and the patient was discharged from the hospital with oral prednisone 10mg daily. He was recommended to follow up with his private rheumatologist and hyperbaric oxygen medicine service at Westchester Medical Center.

**Figure 2:**
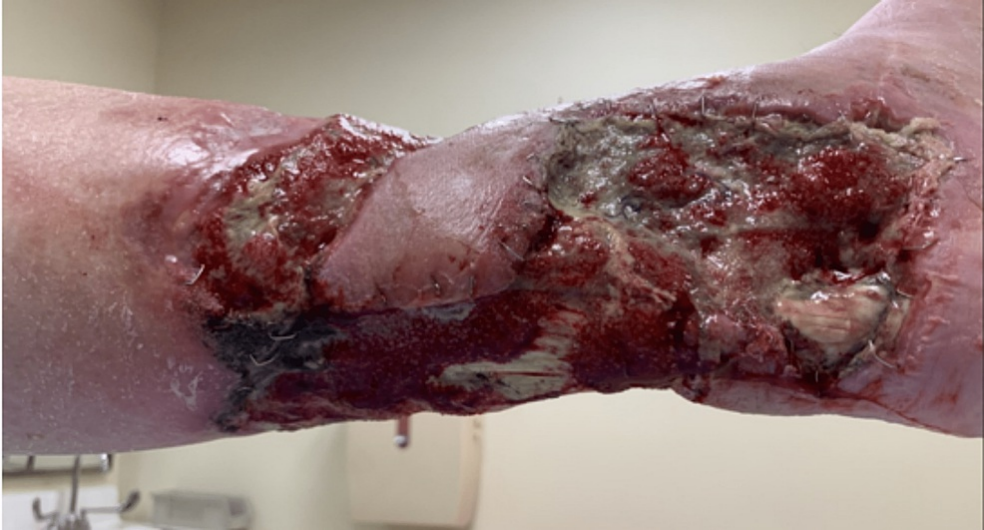
The presentation of the wound during hospitalization in December 2021

In July 2021, the patient was started on methotrexate 20mg weekly by his rheumatologist before presenting to the rheumatology outpatient practice in Westchester in October 2021. The patient had significant alcohol use, placing him at increased risk of methotrexate-induced hepatotoxicity. At that time, methotrexate was discontinued, and he was started on mycophenolate (MMF) 500mg twice a day. The patient reported a good response to MMF because the edges of his wound started to fill up with new skin formation, and he had noticed a reduction in the depth of his wounds as well. However, in December 2021, his wound care physician noted the worsening of the wound that was overlying the Achilles tendon with exposure of the tendon, as shown in Figure 2. Around that time, the patient underwent sharp excisional debridement of left lower extremity ulcers and cadaveric skin graft placement.

**Figure 3:**
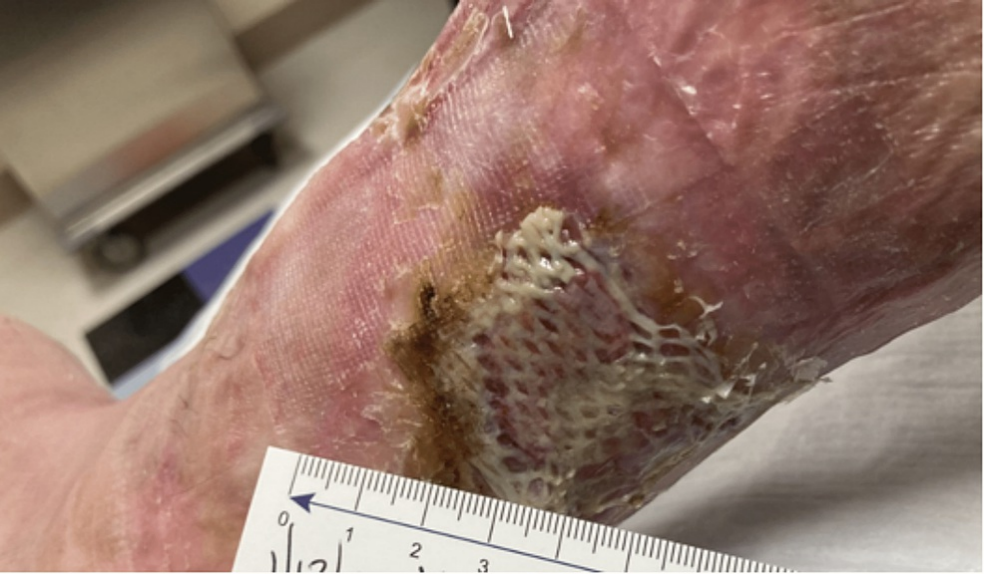
Wound presentation in January 2023

In January 2022, the patient underwent left lower extremity debridement again, along with TheraSkin (cadaver skin graft) application and wound vacuum application. The patient also required debridement of the wound with the application of TheraSkin on the left lower extremity once again in March 2022 as he continued with the MMF and hyperbaric oxygen treatment. In August 2022, the ankle wound along with the exposed tendon area was noted to be healed. The patient received a total dose of MMF of 1000 mg daily. He continues to follow up with the hyperbaric oxygen clinic and wound care clinic. The patient was last seen in an outpatient rheumatology clinic in December 2022. During his visit to the rheumatology clinic, a physical exam showed continued improvement in his lesions; therefore, MMF was decreased to 500mg daily. The patient was last seen in the Burn Center for hyperbaric oxygen therapy in January 2023, and the most recent photo is depicted in Figure 3.

## Discussion

PG can be a challenging condition to manage. In the absence of ample data on the treatment of PG, concrete guidelines for treatment are lacking. Mild to severe PG is usually managed with a local corticosteroid or a calcineurin inhibitor [6-8]. For patients with small and localized lesions, topical tacrolimus 0.3% and intralesional steroids can be utilized. However, treatment with topical therapy alone usually has slow improvements, and relapses are common, so concomitant use of systemic therapy can be used. Systemic steroids remain the most commonly used first-line treatment for the management of PG [9].

The use of infliximab, an antibody against tumor necrosis factor-alpha (TNF-alpha), is supported by placebo-controlled randomized trials. There are case reports and case series that demonstrate that adalimumab, etanercept, golimumab, and certolizumab pegol, which are also TNF-alpha inhibitors, have been shown to have resulted in either complete resolution or partial improvement in symptoms of PG. Other systemic immunosuppressants such as methotrexate, mycophenolate mofetil, sulfasalazine, azathioprine, cyclosporine/tacrolimus, and mycophenolate mofetil are also being used as treatments for PG [9].

Our patient was initially treated with methotrexate and was switched to MMF. MMF is an ethyl ester form of mycophenolic acid with increased bioavailability that is rapidly transformed in the liver into its active metabolite, mycophenolic acid. It selectively inhibits the type 2 isoform of inosine monophosphate dehydrogenase in the de novo purine synthesis pathway [8].

The paucity of literature using MMF in the treatment of pyoderma gangrenosum is notable. A retrospective chart review was conducted by Li et al. in 2013, and 26 patients (one bullous PG and 25 classic PG) were identified for whom MMF was used as a first-line steroid-sparing agent in 11 patients (42.3%), second-line in 14 (53.8%), and third-line in one (3.85%). Overall, 22 patients (84.6%) demonstrated clinical improvement, and 13 patients (50%) achieved complete ulcer healing [7]. The starting dose of MMF was 1 g (24/26 patients) or 2 g (2/26 patients) total daily. Generally, the maintenance dose was 2 g (10/26 patients) or 3 g total daily (13/26 patients). Two patients were maintained on 1 g total daily, and one patient only took MMF for a month at the starting dose before ceasing it. In addition to MMF, patients also received oral prednisolone [7].

Case reports of the efficacy of MMF in treating PG are not very common; however, another case of a 32-year-old woman with a history of systemic lupus erythematosus (SLE) and a two-year history of refractory PG was noted in 1998 by Nousari et al. The patient presented with a lesion in her left pretibial area, which eroded to expose underlying tendons and muscles, resulting in a unilateral foot drop. During this two-year period, the patient’s lesion did not respond to prednisone as well as prednisone in combination with azathioprine, 2.5 mg/kg per day; dapsone, 100 mg/d; oral cyclophosphamide, 2 mg/kg per day; and intralesional triamcinolone acetonide injections. MMF 500mg twice a day was added to the patient's existing therapeutic regimen of oral microemulsion cyclosporine, 5 mg/kg per day, and prednisone, 1 mg/kg per day, and by week 14, the PG lesion was almost completely healed [8]. Mycophenolate so far seems to be a promising drug for immunologically mediated skin diseases like pyoderma gangrenosum, as it was effective for our patient discussed in this case report.

The concomitant use of hyperbaric oxygen therapy (HBOT) is also significant in this case. Since the patient was started on MMF, he has continued to receive hyperbaric oxygen treatment multiple times a month. Case reports of the use of HBOT for treating PG remain scarce. A case of a 65-year-old man from Korea who developed PG associated with ulcerative colitis was reported by Seo et al. After three months of treatment with hyperbaric oxygen therapy, the patient’s PG lesions completely resolved without any oral medication treatment for PG. HBOT is performed in a closed hyperbaric oxygen chamber at about two to three atmospheres with 100% oxygen and facilitates wound healing. HBOT increases the amount of dissolved oxygen in the blood. It inhibits vasoconstriction, promotes angiogenesis, and also has some bactericidal effects. HBOT can potentially have some adverse effects, such as reversible myopia and equalization problems due to pressure differences in either one or both ears [10].

## Conclusions

Treating PG requires a multidisciplinary approach. Based on this case report, MMF should be considered as one of the treatment options for patients with PG, especially those who are refractory to the first line of drugs. Treatments such as hyperbaric oxygen therapy can be used to improve healing and patients’ quality of life.
